# A Selection of Reliable Reference Genes for Gene Expression Analysis in the Female and Male Flowers of *Salix suchowensis*

**DOI:** 10.3390/plants11050647

**Published:** 2022-02-27

**Authors:** Fangwei Zhou, Yingnan Chen, Huaitong Wu, Tongming Yin

**Affiliations:** Key Laboratory for Tree Breeding and Germplasm Improvement, Southern Modern Forestry Collaborative Innovation Center, College of Forestry, Nanjing Forestry University, Nanjing 210037, China; zhoufangwei@njfu.edu.cn (F.Z.); chenyingnan@njfu.edu.cn (Y.C.); tmyin@njfu.com.cn (T.Y.)

**Keywords:** *Salix*, flower, reference gene, *ACT*, *DnaJ*

## Abstract

*Salix* is a dioecious plant. Research on the molecular regulation mechanism of male and female inflorescence differentiation and development is necessary to analyze sex differentiation in the willow and the underlying mechanisms of unisexual flower development. However, at present, there are no reference genes suitable for stable expression in the process of willow inflorescence development. In this study, *Salix suchowensis* was used as the research material, nine candidate reference genes (*α-TUB1*, *α-TUB2*, *ACT*, *H2A*, *DnaJ*, *CDC2*, *GAPDH*, *TIP41*, *β-TUB*) were selected, and qRT-PCR technology was used to detect the expression of each candidate reference gene in female and male flowers at different developmental stages and using five algorithms (geNorm, Normfinder, Delta Ct, BestKeeper, and RefFinder) to comprehensively evaluate the stability of candidate reference genes. The results showed that *ACT* and *DnaJ* were stably expressed in all samples and could be used as reference genes. In addition, the reliability of the screening results was further verified via an expression pattern analysis of the *CFS* gene that encodes flower specific transcription factor in different samples. The stable reference genes selected in this study provide the basis for future research on the expression analysis of functional genes related to the development of male and female flowers of *S. suchowensis*.

## 1. Introduction

*Salix* is widely distributed in the northern hemisphere. Due to its rapid growth, easy asexual reproduction, and strong environmental adaptability, it is often used as a landscaping and economic timber tree species [1,2,3]. The reproductive characteristics of willow are dioecious. The female willow tree has good material qualities, and its growth performance is generally better than the male plant and so the elite clones of willow trees that are selected and promoted are mainly female plants. The gender dimorphism of willow trees is mainly reflected in the differences in the flower organs [4]. After the female flower is pollinated, the capsules mature and produce catkins that are then released into the air and can cause breathing difficulties in humans [5]. After the male flower matures, it produces a large amount of pollen. The pollen grains are small and light and can easily be spread by wind, acting as a potential allergen upon contact and inhalation in humans [6,7]. These characteristics restrict the cultivation and breeding of willows [8]. Whilst considering the strong ecological and economic benefits of willow trees, there is an urgent need to control the seasonal pollution caused by catkins and pollen. The cloning of genes that regulate the differentiation and development of flower organs in the willow tree can help the cultivation of new varieties through gene editing and other technologies to ultimately reduce the production of catkins and pollen. *Salix suchowensis* is a small shrub native to China, which can flower in the same year after cutting. It has a short individual size and short generation cycle, facilitating large-scale field experiments [9]. *S. suchowensis* is the first species in the genus *Salix* to have its whole genome sequenced. It can therefore be used as a model species for forest tree functional gene mining [9,10].

The real-time quantitative PCR (qRT-PCR) has an extremely high sensitivity, specificity, reproducibility, and is the most basic method for studying gene transcription and regulation [11,12]. In order to ensure the accuracy and reliability of qRT-PCR data, suitable reference genes must be used for standardization. Reference genes are a class of genes that are stably expressed in different experimental conditions, different varieties, different stress treatments, and different tissues [13]. Choosing the appropriate reference gene is the primary factor to ensure the accuracy of an experiment [14]. For example, actin (*ACT*), glyceraldehyde-3-phosphate dehydrogenase (*GAPDH*), tubulin beta chain (*β-TUB*), chaperone protein (*DnaJ*), and others are frequently used as reference genes in different species [15,16]. However, there is currently no reference gene in *Salix* that is not restricted by conditions and can be stably expressed under different developmental periods and biotic/abiotic stress conditions [17]. Therefore, it is of great significance for gene quantitative analysis to screen for reference genes that are stably expressed under different test conditions.

Studies have reported that in *Salix viminalis*, reference genes such as Type 2A phosphatase Activator (*TIP41*) and Cyclin Dependent Kinase-putative (*CDC2*) are stably expressed in roots under different stress conditions [16]. In leaves, *TIP41* is the most stable [16]. In *Salix matsudana,* alpha-tubulin2 (*α-TUB2*) and chaperone protein DnaJ 49 (*DnaJ*) are reference genes that are stably expressed in different tissues under different stress conditions [15]. However, the reference genes identified in *Salix psammophila* are completely different in different tissues and under different stress conditions [18]. According to the results of previous studies, the most stable reference genes of the willow tree are not the same in different varieties, different tissues, different developmental periods, and different stress conditions [15,16,18]. However, there is currently no one reference gene used for research in the development of male and female flowers in *S. suchowensis*. Unstable reference genes can cause significant errors in the data in different species and under different experimental conditions, thereby affecting the accuracy of the target gene detection [17]. Therefore, screening reference genes that are stably expressed at different stages of male flower development in *S. suchowensis* is key for the accuracy of gene quantitative analysis.

In this study, female, and male flowers of *S. suchowensis* at different developmental stages were selected as experimental materials: T1 (differentiation stage), T2 (dormancy stage), T3 (early flowering stage), and T4 (full flowering stage). Nine candidate internal reference genes (alpha-tubulin1 (*α-TUB1*), alpha-tubulin2 (*α-TUB2*), actin (*ACT*), histone H2A (*H2A*), chaperone protein DnaJ 49 (*DnaJ*), cyclin-dependent (*CDC2*), glyceraldehyde-3-phosphate dehydrogenase (*GAPDH*), Type 2A phosphatase activator (*TIP41*), and tubulin beta chain (*β-TUB*)) that are frequently used in different species and whose stability has been verified in different tissues were selected [19,20]. In the genome of *S. suchowensis*, the full-length sequences of nine candidate reference genes were obtained through homologous sequence alignment. The expression analysis of each reference gene in female and male flowers at different developmental stages was performed by qRT-PCR. Five statistical algorithms (GeNorm, NormFinder, Delta Ct (ΔCt), BestKeeper and RefFinder) were used to analyze the stability of the expression of nine candidate genes and to determine the most stable reference gene. Cop/fus-specific (*CFS*) gene, which is a homologous gene of *AtMYB21* and encodes the flower specific transcription factor [21], was selected to verify the stability of the expression of the selected reference gene. This study, therefore, recommends reference genes for facilitating qRT-PCR analysis in female and male flower bud development in the willow tree. This approach also offers the potential to study the regulatory mechanisms of sex differentiation and to explore the regulatory effects of target genes in male and female willow flower buds. Moreover, this effective genetic tool may be used to create a new willow germplasm.

## 2. Results

### 2.1. RNA Quality and Primer Specificity Analysis

**Figure 1:**
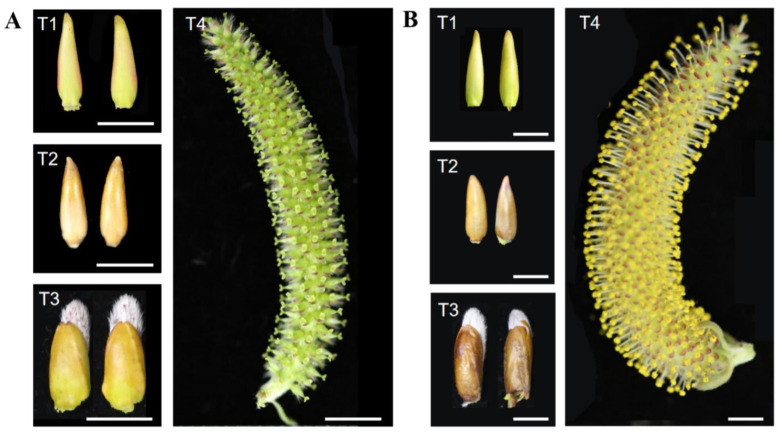
The morphology of *S. suchowensis* female (**A**) and male (**B**) flowers at the four developmental stages. T1 (differentiation stage), T2 (dormancy stage), T3 (initial flowering stage), and T4 (full flowering stage). Bar = 0.5 mm.

In this study, NanoDrop™ One (Thermo Scientific, New York, NY, USA) and 1% agarose gel electrophoresis were used to detect the total RNA quality of eight female and male flowers at different developmental stages in *S. suchowensis* (Figure 1). The OD 260/280 value of the RNA obtained was between 1.8 and 2.1 (Figure S1A). Agarose gel electrophoresis showed that the RNA quality was good and the 28S and 18S ribosomal RNA subunit bands were clear and strong, indicating that there was no significant degradation (Figure S1B), and the RNA quality fully met the needs of subsequent experiments. The candidate gene primers fully amplified all nine reference genes producing products 100–250 bp in length (Figure S2). The qRT-PCR melting curve demonstrated that each reference gene produced only a single melting peak without other heteropeaks (Figure S3) and the curve overlap between the repeated samples was good, indicating that the specificity of the reference gene primer amplification was high (Figure S4). In addition, this study constructed standard curves for the reference genes, and the results showed that the amplification efficiency (E) of nine pairs of primers were all between 88.66 and 106.62%. The differences in the R^2^ values were small and were both greater than 0.96 (Table 1). These results suggest that the amplification efficiency and product specificity of each reference gene met the conditions for the qRT-PCR and that the stability of the reference genes could be further evaluated.

### 2.2. Reference Gene Expression Levels 

**Figure 2:**
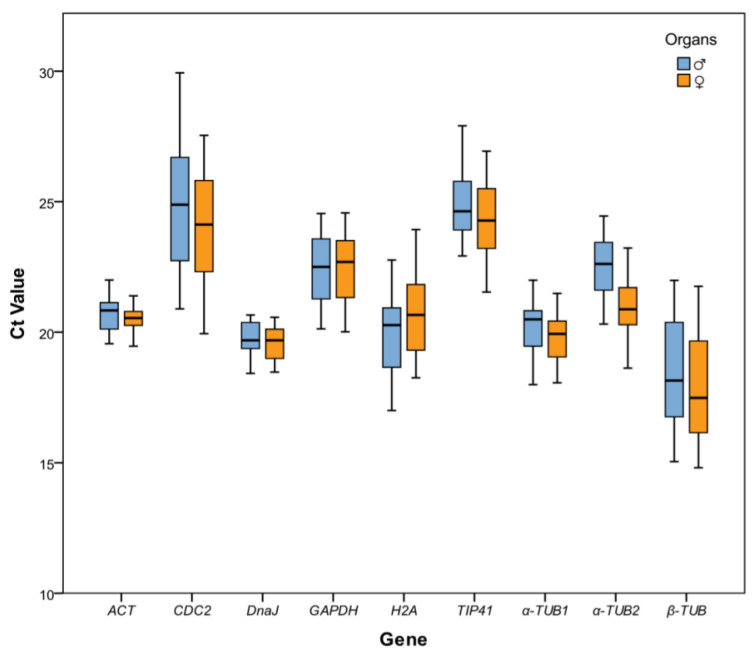
The Ct value distribution of the nine candidate reference genes in eight samples. The data are the Ct values of every single gene in the female or male flowers in all experimental groups. The boxplot is the concentrated range of the Ct values, and the horizontal line is the median. The upper and lower side lines of the boxplot are the upper quartile and the lower quartile, respectively. The upper and lower end lines of the box are the maximum and minimum data, respectively.

The Ct value was used to estimate the gene expression level. There were significant differences in the Ct value ranges of the nine candidate reference genes in the female and male flowers at different developmental stages. The smaller the Ct value, the higher the gene expression level, and a Ct value of the reference gene that is too high (>30) or too low (<10) is not suitable for subsequent analysis of the target gene [22]. The minimum Ct value of *β-TUB* was 15.17 and the maximum Ct value of *CDC2* was 29.51, which all met the Ct value range required by the qRT-PCR (Figure 2). Among all the candidate reference genes, the highest average expression was observed in the *β-TUB* gene, with an average Ct value of 18.26. The lowest average expression was in the *CDC2* gene, with an average Ct value of 24.46. The Ct value of the *DnaJ* gene was 18.46–20.64, which showed the lowest variation in the expression level among the nine candidate reference genes. However, the expression level variation of the *CDC2* gene was the highest, with a Ct value of 20.09–29.51. These results show that variation in the expression level of the candidate reference genes in different tissues at different developmental stages was significantly different. None of them had a completely stable expression pattern across all samples. Therefore, five different statistical algorithms were further used to evaluate the expression stability of the nine reference genes in male and female flowers at different developmental stages.

### 2.3. Expression Stability Analysis of the Candidate Reference Genes

Four algorithms (geNorm, NormFinder, ΔCt, and BestKeeper) were used to analyze and rank the expression stability of the nine candidate reference genes. The results were then comprehensively analyzed with RefFinder to rank the overall stability of the candidate reference genes in different samples and to determine the efficiency of using these reference genes for male and female flowers at different developmental stages.

#### 2.3.1. geNorm Analysis

**Figure 3:**
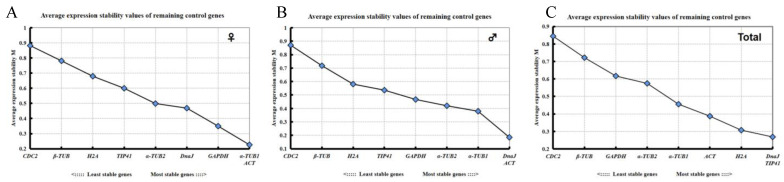
M values of the nine candidate reference genes. geNorm algorithm analysis of the stability of candidate reference genes in female flowers (**A**), male flowers (**B**), and all samples (**C**).

geNorm was used to analyze the nine candidate reference genes. The results showed that the M values of all reference genes in different flowers were less than 1.5 (Figure 3), inferring stability. The most stable reference genes in the female flowers were *ACT* and *α-TUB1*, while the most stable reference genes in male flowers were *ACT* and *DnaJ*. In the comprehensive analysis of the male and female flowers at different developmental stages, *TIP41* and *DnaJ* were the most stable reference genes. The order of expression stability from high to low was: *TIP41* = *DnaJ* > *H2A* > *ACT* > *α-TUB1* > *α-TUB2* > *GAPDH* > *β-TUB* > *CDC2*.

**Figure 4:**
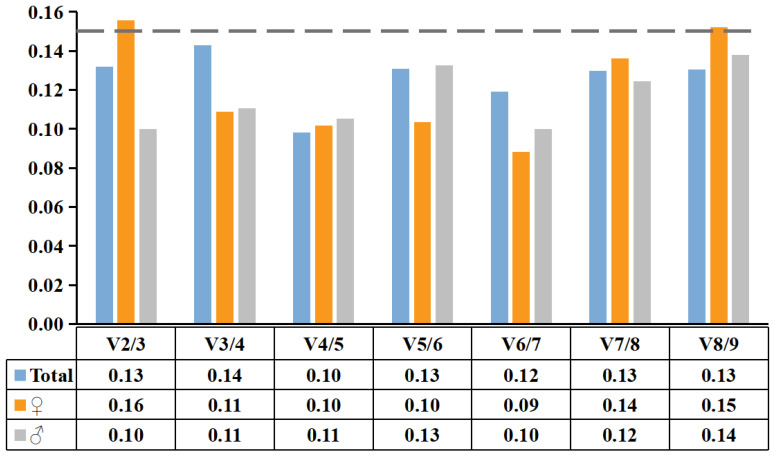
Determination of the optimal number of reference genes by pairwise variation (V_n/n+1_). The V_n/n+1_ value of reference genes in male or female flowers was analyzed by geNorm to determine the most suitable number of reference genes under different conditions. The critical value of V_n/n+1_ is 0.15. When V_n/n+1_ was less than 0.15, the n reference genes were selected as the most suitable.

In addition, the geNorm software could also determine the optimal number of reference genes required by calculating the pairwise variation (V_n/n+1_) of candidate reference genes in female and male flowers at different developmental stages of *S. suchowensis* (Figure 4). When V_n/n+1_ < 0.15, the optimal number of reference genes was n, and there was no need to introduce the n + 1 reference gene. V_2/3_ = 0.16> 0.15 in the female flowers of the four developmental stages. When the third reference gene was added V_3/4_ = 0.11 < 0.15, indicating that three reference genes were required for accurate normalization. For male flowers, two reference genes (*ACT* and *DnaJ*) could be used for accurate normalization because V_2/3_ = 0.10 < 0.15. When all the flowers were analyzed, V_2/3_ = 0.13 < 0.15, indicating that using two reference genes (*ACT* and *DnaJ*) was the best standardization method.

#### 2.3.2. NormFinder Analysis

NormFinder obtains the most stable reference gene according to the S value (stable value) [23]. Stability analysis of the nine reference genes in the female and male flowers of *S. suchowensis* at different developmental stages (Table 2) showed that the most stable reference genes in the female and male flowers were *DnaJ* (S value = 0.022) and *ACT* (S value = 0.038), respectively. A comprehensive analysis of all the samples demonstrated that the overall most stable reference gene was *ACT* and the order of stability from high to low was *ACT* > *DnaJ* > *TIP41* > *H2A* > *α-TUB1* > *β-TUB* > *α-TUB2* > *GAPDH* > *CDC2*. In most cases, the most unstable gene was *CDC2*.

#### 2.3.3. ΔCt Analysis

ΔCt analyzes the changes in the relative expression of gene pairs in each sample, which is similar to geNorm to some extent [24]. The ΔCt method was further used to determine the expression stability of the nine candidate reference genes. *ACT* and *α-TUB1* were the most stable reference genes expressed in female flowers at different developmental stages. *ACT* and *DnaJ* were most stably expressed in male flowers. The ΔCt analysis results showed that the stability of the reference genes in all samples from high to low was *ACT* > *DnaJ* > *TIP41* > *α-TUB2* > *H2A* > *α-TUB1* > *GAPDH* > *β-TUB* > *CDC2* (Table 3). These results suggested that *ACT* and *DnaJ* were the ideal reference genes.

#### 2.3.4. BestKeeper Analysis

BesterKeeper software can directly analyze the Ct value of each reference gene obtained by the qRT-PCR to identify the correlation coefficient (R^2^), standard deviation (SD), and coefficient of variation (CV) [25]. The reference genes with sufficient stability were identified by comparing these values. The larger the correlation coefficient, the smaller the value of CV ± SD, and the better the stability of the reference gene, and vice versa. When SD > 1, it was considered that the expression of the reference genewas unstable. The BestKeeper program was used to analyze the stability of the nine candidate reference genes. The results showed that the two reference genes with the most stable expression were *ACT* and *DnaJ*, with CV ± SD values of 2.42 ± 0.50 and 2.85 ± 0.56, respectively (Table 4). Moreover, the most unstable genes were *β-TUB* and *CDC2*. According to the SD and CV of the Ct value, each reference gene stability was ranked from high to low in the following order: *ACT* > *DnaJ* > *α-TUB1* > *GAPDH* > *α-TUB2* > *TIP41* > *H2A* > *β-TUB* > *CDC2*. 

#### 2.3.5. RefFinder Analysis

**Figure 5:**
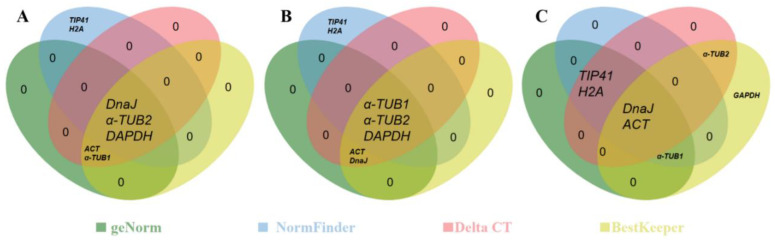
The top five most stable reference genes generated by geNorm, NormFinder, Delta-Ct, and BestKeeper. The green, blue, pink, and yellow circles contain the top five most stable reference genes identified in (**A**) female or (**B**) male flowers and (**C**) all samples by geNorm, NormFinder Delta-Ct, and BestKeeper, respectively. The genes in the overlapping regions were identified as the top five most stable reference genes by more than one algorithm.

There were some differences in the analysis results of the nine candidate reference genes between the four algorithms [26]. Therefore, the comprehensive analysis tool RefFinder was further used to calculate the geometric average of the analysis results of the four algorithms in order to evaluate the stability of the nine candidate reference genes more effectively. The results showed that the two genes with the most stable expression were *ACT* and *DnaJ* (Table 5). The expression stability of these two genes in different flowers ranked in the top five among the four algorithms (at least three) (Figure 5). In addition, a comprehensive analysis of the reference genes with the lowest expression stability showed that *β-TUB* and *CDC2* were the two most unstable genes.

### 2.4. Reference Gene Stability Verification 

**Figure 6:**
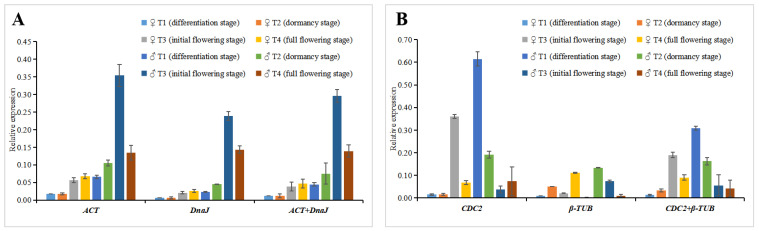
*CFS* gene expression pattern analysis. The expression of the *CFS* gene in male and female flowers was analyzed by qRT-PCR. (**A**) The data were normalized with the most stable reference genes *ACT* and *DnaJ* identified. (**B**) The most unstable, *β-TUB* and *CDC2*, were used as controls, using the 2^−ΔΔCt^ method to analyze gene expression.

In order to verify the stability of the two reference genes *ACT* and *DnaJ* identified by the above evaluation, the expression patterns of the *CFS* gene related to flower development in female and male flowers of *S. suchowensis* at different developmental stages were analyzed. The two most unstable reference genes (*β-TUB* and *CDC2*) were used as controls. Following *CFS* gene expression profile standardization, the results showed that when the stable reference genes *ACT* and *DnaJ* were used for standardization, the relative expression of the target gene had the same trend (Figure 6). However, when the two unstable reference genes (*β-TUB* and *CDC2)* were used to normalize the data, the *CFS* gene expression results were inconsistent with the previous results (Figure 6). Therefore, the qRT-PCR results of the *CFS* gene expression verified that *ACT* and *DnaJ* have good stability in the male and female flowers of *S. suchowensis* at different developmental stages. In addition, previous studies have shown that using two or more reference genes to standardize data is more reliable than using one [23,24]. Therefore, both *ACT* and *DnaJ* were selected as stable reference genes in female and male *S. suchowensis* flowers.

## 3. Discussion

Flowering is a qualitative change process in the growth and development of higher plants and the development of floral organs has always been of interest in plant research [27]. The willow is a dioecious tree, and the gender differences are primarily reflected in the variation of the flower organ morphology [28,29]. There are no pistil primordia in the male flowers and no stamen primordia in the female flowers. The availability of the high-quality willow genome has encouraged research on the regulation mechanisms of the differentiation and development of the willow inflorescences [9]. At present, many studies related to the regulation of sex differentiation in the willow have predicted the candidate genes of sex regulation in the sex determination interval [30,31,32]. Through RNA-seq analysis, it was found that genes located in the sex-determining region show unique and flower-biased expression patterns [30,31,32]. Therefore, in the follow-up research on sex differentiation or flower development of the willow, in order to make the quantitative experimental results of related functional gene expression analysis more accurate, a stable expression reference gene is needed to normalize the data. 

*Salix suchowensis* is a rapidly growing diploid shrub willow and can blossom in the year of cutting or sowing. Its genome has been fully sequenced and can be used as a model species for forest tree functional gene mining [9]. Therefore, we selected *S. suchowensis* to mine for stably expressed reference genes in male and female flowers at different developmental stages. Flower development is an important integrated development process in the life cycle of angiosperms [33]. In this study, the four important stages of male and female flower development of *S. suchowensis* were selected (T1–T4) to verify the stability of reference genes.

Previously, due to the cumbersome steps, heavy workload, and small application scope of discovering new reference genes, the development of new reference genes has been limited [33]. At present, many statistical algorithms have been developed to identify the most stable reference genes for the standardization of target gene expression [34,35]. In this study, the five most commonly used reference gene stability evaluation algorithms (GeNorm, NormFinder, ΔCt, BestKeeper, and RefFinder) were used to comprehensively evaluate the expression stability of the nine candidate reference genes in male and female flowers at different developmental stages. The results showed that despite the different calculation methods and principles of each program, the stability results were essentially the same. However, some differences were still observed. In this study, we compared the top five reference genes in the analysis results of different programs and found that GeNorm and NormFinder had the same expression stability results of candidate reference genes in all samples. In order to avoid errors caused by different algorithms, we used RefFinder to calculate a geometric average of the ranking of the four software analysis results and comprehensively analyzed the stability of the nine candidate reference genes.

According to the four algorithms and the comprehensive evaluation of RefFinder, *ACT*, and *DnaJ* were the most stable reference genes in both the male and female flowers of *S. suchowensis* at different developmental stages. *ACT* is actin and one of the three main components of the cytoskeleton [36]. The growth of pollen tubes in male flowers requires the coordination of the cytoskeletal dynamics and apical secretion [37]. In the female gametes of flowering plants, sperm nucleus migration is controlled by the continuous inward movement of actin filaments for successful fertilization [38]. *ACT* is a protein with a highly conserved amino acid sequence. It displays almost no changes throughout the evolution of plants, and the expression of *ACT* in various tissues is high and consistent [39]. Therefore, *ACT* is the most commonly used reference gene for qRT-PCR. It is widely used in both model plants such as *Arabidopsis* and non-model species or plants without a reference genome, such as *Japanese gentian* [40,41,42,43,44,45]. 

*DnaJ* is a chaperone protein. Membraneless organelles contain a wide range of chaperone proteins, indicating that they play an important role in regulating the assembly and maintenance of membraneless organelles and biological functions [46]. A recent study shows that *DnaJ* is a stably expressed reference gene in different tissues of willow under different stress conditions [15]. Our study confirms previous research results. In addition, previous studies have shown that using only one reference gene often produces some errors in the qRT-PCR results and the combination of two or more reference genes can increase the accuracy of the results [23,24]. In this study, geNorm results showed that the V_2/3_ value was less than 0.15 in both male and female flowers of *S. suchowensis* at different developmental stages, suggesting that the combination of two reference genes (*ACT* and *DnaJ*) can achieve stable normalization. Furthermore, the expression pattern of the target gene (*CFS*) demonstrated the reliability of the combination of *ACT* and *DnaJ* as reference genes for both female and male flowers at different developmental stages of *S. suchowensis*. For the first time, this study screened out stably expressed reference genes in female and male flowers of *S. suchowensis*, providing a scientific basis for subsequent studies on the quantitative expression of functional genes related to flower bud development in *S. suchowensis.*

## 4. Materials and Methods

### 4.1. Plant Material

*Salix suchowensis* was grown at the Baima Base of Nanjing Forestry University, Nanjing, China (32° N, 118° W). Healthy female and male plants (three each) that were the same height and at the same developmental stage were randomly selected for sample collection. From September 2020 to March 2021, four stages of male and female flower development were collected: T1 (differentiation stage), T2 (dormancy stage), T3 (initial flowering stage), and T4 (full flowering stage) (Figure 1). Three female or male flowers from each tree in each developmental period were collected into a centrifuge, flash-frozen with liquid nitrogen, and stored at −80 °C until further use.

### 4.2. Extraction of Total RNA from Plant Tissues and cDNA Aynthesis 

The RNAprep Pure Polysaccharide Polyphenol Plant Total RNA Extraction Kit (TIANGEN, BeiJing, China) was used to separate total RNA from female or male flowers at different developmental stages. NanoDrop™ One (Thermo Scientific, New York, NY, USA) and 1% agarose gel electrophoresis were used to measure the RNA concentration and quality, respectively. The cDNA reverse transcription reaction system for qRT-PCR analysis was prepared according to the One-Step gDNA Removal and cDNA Synthesis SuperMix (TransGen Biotech, BeiJing, China) instructions and 1 µg of total RNA was used in the 20 μL cDNA reverse transcription reaction system. After the reaction was completed, the samples were stored at −20 °C for future use.

### 4.3. Selection of Reference Genes and Primer Design

Nine candidate reference genes (*α-TUB1*, *ACT*, *α-TUB2*, *H2A*, *DnaJ*, *CDC2*, *GAPDH*, *TIP41*, *β-TUB*) and one target gene (*CFS*) (Table 1) were selected. Manual BLAST search in the *S. suchowensis* genome, the full-length transcription sequences of each candidate gene were determined [9]. Primer Premier 5.0 was used to design primers based on the full-length transcription sequence of the gene [47]. The standards were as follows: GC content 45–65%, optimal Tm 58–61 °C, primer length 18–22 bp, and amplicon length 100–250 bp. Primer specificity was identified using BLAST in the whole genome of *S. suchowensis*. Primers were synthesized by Sangong Bioengineering Shanghai Co., Ltd., China (Table 1). Preliminary screening of the primers was performed using a PCR and 1% agarose gel electrophoresis to observe the product specificity with the presence of correct PCR bands. Primers producing the correct band size, good band specificity, and no primer dimers were further selected for qRT-PCR analysis. The primers with a single peak map, no heteropeak, and no peak in the negative control were selected as the final primers.

### 4.4. qRT-PCR of Candidate Reference Genes

The qRT-PCR was performed using Applied Biosystems StepOne (Thermo Fisher Scientific, USA). Each reaction contained 4 pM of each forward and reverse primer, 2 µL of template cDNA diluted in different multiples, 10 µL PowerUp™ SYBR™ Green Master Mix (Thermo Scientific, New York, NY, USA), and ddH_2_O topped up to a total volume of 20 µL. The reactions were performed on the 7500 Fast Real-Time PCR System (Applied Biosystems, New York, NY, USA). The qRT-PCR reaction conditions were as follows: 95 °C for 3 min, followed by 40 cycles of 95 °C for 15 s, 60 °C for 15 s, and 72 °C for 30 s. At the end of each experiment, the melt curve analysis was performed at 55–95 °C with 0.3 °C increments for 60 s using the default parameters. All analyses were performed in three biological replicates

### 4.5. Establishment of Reference Gene Primer Standard Curve

A standard curve for each pair of primers of each reference gene was generated by calculating the amplification efficiency of the corresponding primers. The reverse-transcribed cDNA was diluted into five gradient concentrations (1, 1/5, 1/25, 1/125, 1/625) as the template for establishing the standard curve. ddH_2_O was used as a negative control template to detect reagents or contamination during the experiment. All samples were repeated in triplicate to ensure the credibility of the experimental data. qRT-PCR was performed using Applied Biosystems StepOne PCR System (Thermo Fisher Scientific, USA) to obtain the cycle threshold (Ct) value of each candidate reference gene at different template dilution concentrations. A standard curve was generated with the log value on the x-axis and the Ct value on the y-axis to obtain the slope (K) and correlation coefficient (R^2^) with the following formula: E = [5^(1/−K)^ − 1] × 100%. The amplification efficiency of the nine candidate reference genes was calculated, and the amplification efficiency of the selected primers was required to be between 80 and 120% by the qRT-PCR [48].

### 4.6. Data Processing

The geNorm algorithm was used as a pairwise comparison approach to calculate the gene expression stability (M value) [23]. The original Ct values were converted to 2^−∆Ct^ values (delta Ct = original Ct value − the lowest Ct value in each group) used for stability analysis. The stability of genes was measured according to the average degree of the M value variation. When the M value is lower than 1.5, the lower the M value, the higher the stability and vice versa. The algorithm can also calculate the average pairwise variation (V value) of the normalization factor after the introduction of a new reference gene and can determine the number of optimal reference genes required according to the V_n/n+1_ value. When the paired variation value V_n/n+1_ < 0.15, the most suitable genes of the reference gene combination are the top n in the high stability ranking [35].

The NormFinder algorithm adopts the model-based method and also calculates the stable value of reference gene expression through the 2^−∆Ct^ method and then selects the most appropriate reference gene according to the stable value. The reference gene with the lowest stable value is the most appropriate [24].

The ΔCt algorithm identifies stable reference genes by comparing the relative expression of gene pairs in each sample. Two genes are considered to be stably expressed in these samples if the ΔCt value between the two genes remains constant. However, if the ΔCt value fluctuates, the expression of one or both genes is unstable. The third, fourth, and fifth genes can then be introduced into the comparison to provide more information and allow for gene ranking. Finally, the appropriate reference genes can be selected according to the experimental needs [49].

The BestKeeper algorithm uses repeated paired correlation analysis and regression analysis to calculate the standard deviation (SD) and coefficient of variation (CV) of the Ct value. The size of each value can be compared to finally determine the reference gene with better stability [25].

The RefFinder algorithm can comprehensively sort the results obtained from the analysis of four algorithms, assign an appropriate weight to each candidate reference gene, and calculate the geometric average of its weight to obtain the overall total ranking and comprehensively evaluate the stability of candidate genes [26].

### 4.7. Reference Gene Verification

The target *CFS* gene [50], which encodes flower specific transcription factor, was used to verify the stability of the selected reference genes. The primer sequences of the target gene are shown in Table 1 and the qRT-PCR procedure was the same as described above. The gene expression was analyzed using the 2^−ΔΔCt^ method [51,52,53].

## Figures and Tables

**Table 1:** Analysis of candidate reference genes, primer sequences, and qRT-PCR amplification characteristics.

Gene	Gene Description	Locus Name	Primer Sequence F/R(5′-3′)	Product Size (bp)	Efficiency (%)	R^2^
*α*-*TUB1*	alpha-tubulin1	EVM0011215.1	CTCTTGGAGCGTTTGTCAGT	175	100.25	0.968
			TCGTAGATGGCTTCATTGTC			
*α*-*TUB2*	alpha-tubulin2	EVM0036214.1	TTTCAAGTGCGGTATCAACT	250	96.45	0.981
			CTTCCTCGTAGTCTTTCTCAA			
*β*-*TUB*	β-Tubulin	EVM0022575.1	AATGCGGCAACCAAATAGGA	164	86.66	0.996
			TAGAACAGCACGAGGGACAA			
*DnaJ*	chaperone protein DnaJ 49	EVM0005127.1	GAGCCTATTTATGCCCTTTC	162	95.45	0.990
			GACGTAGTCCTTATCCACCTT			
*CDC2*	Cyclin Dependent Kinase-putative	EVM0002694.1	GATGTATGGTCAGTGGGATG	158	106.62	0.983
			TTGAAGTCAGGCAAAGAAGT			
*ACT*	actin	EVM0032804.1	CCAAGGAGGCTGCTGGTAAT	189	98.90	0.993
			CCAAGGCCACCATGTCTGTC			
*TIP41*	Type 2A phosphatase activator	EVM0041976.1	GCTAATGAGATTAAGGGACA	142	101.06	0.985
			TGAAGCAGAGTCAGCAGGAT			
*GAPDH*	glyceraldehyde-3-phosphate dehydrogenase	EVM0035312.1	CGAAGAAGGGCATTACAGCA	121	96.08	0.970
			GAGCATCGGAAGTCAACAGA			
*H2A*	histone H2A	EVM0033846.1	GCAAGTATGCTGAGCGTGTT	123	97.91	0.985
			TACGGGTCTTCTTGTTGTCTC			
Target gene						
*CFS*	cop/fus-specific	EVM0013916.1	AAGTTGGGAAACAGGTGGTC	194	97.57	0.993
			GTCTCCATTTGGTCGGTTGT			

**Table 2:** NormFinder analysis of the S value of the nine candidate reference genes.

Rank		1	2	3	4	5	6	7	8	9
♀	Gene	*DnaJ*	*α-TUB2*	*TIP41*	*GAPDH*	*H2A*	*α-TUB1*	*β-TUB*	*ACT*	*CDC2*
	Stability	0.022	0.023	0.257	0.315	0.377	0.573	0.612	0.624	0.823
♂	Gene	*ACT*	*α-TUB2*	*α-TUB1*	*TIP41*	*H2A*	*DnaJ*	*GAPDH*	*β-TUB*	*CDC2*
	Stability	0.038	0.074	0.213	0.215	0.303	0.536	0.563	0.631	0.951
Total	Gene	*ACT*	*DnaJ*	*TIP41*	*H2A*	*α-TUB1*	*β-TUB*	*α-TUB2*	*GAPDH*	*CDC2*
	Stability	0.068	0.093	0.174	0.259	0.283	0.575	0.585	0.616	0.861

**Table 3:** ΔCt analysis of the Genes Average of STEDV of the nine candidate reference genes.

Rank		1	2	3	4	5	6	7	8	9
♀	Gene	*ACT*	*α-TUB1*	*TIP41*	*GAPDH*	*H2A*	*DnaJ*	*β-TUB*	*α-TUB2*	*CDC2*
	STEDV	0.68	0.68	0.75	0.76	0.83	0.96	1.01	1.03	1.23
♂	Gene	*ACT*	*DnaJ*	*α-TUB1*	*TIP41*	*H2A*	*α-TUB2*	*GAPDH*	*β-TUB*	*CDC2*
	STEDV	0.63	0.66	0.68	0.77	0.79	0.90	0.94	1.06	1.40
Total	Gene	*ACT*	*DnaJ*	*TIP41*	*α-TUB2*	*H2A*	*α-TUB1*	*GAPDH*	*β-TUB*	*CDC2*
	STEDV	0.64	0.65	0.68	0.72	0.73	0.94	0.98	0.98	1.27

**Table 4:** BestKeeper calculation of the SD and CV of the Ct values of each reference gene.

Rank	♀			♂			Total		
	Gene	SD	CV	Gene	SD	CV	Gene	SD	CV
1	*ACT*	0.42	2.05	*ACT*	0.55	*2.68*	*ACT*	0.50	2.42
2	*DnaJ*	0.56	2.84	*DnaJ*	0.56	2.86	*DnaJ*	0.56	2.85
3	*α-TUB1*	0.77	3.88	*α-TUB1*	0.89	4.40	*α-TUB1*	0.84	4.21
4	*α-TUB2*	1.01	4.84	*α-TUB2*	1.05	4.67	*GAPDH*	1.11	4.94
5	*GAPDH*	1.05	4.64	*GAPDH*	1.17	5.21	*α-TUB2*	1.22	5.63
6	*TIP41*	1.32	5.44	*H2A*	1.19	5.94	*TIP41*	1.30	5.28
7	*H2A*	1.47	7.07	*TIP41*	1.24	4.95	*H2A*	1.34	6.57
8	*β-TUB*	1.85	10.28	*β-TUB*	1.88	10.16	*β-TUB*	1.88	10.28
9	*CDC2*	2.08	8.66	*CDC2*	2.16	8.64	*CDC2*	2.15	8.8

**Table 5:** RefFinder comprehensive ranking of the expression stability of the nine candidate reference genes.

Method	1	2	3	4	5	6	7	8	9
Ranking order under female flower bud (Better-Good-Average)									
geNorm	*ACT/* *α-TUB1*		*GAPDH*	*DnaJ*	*α-TUB2*	*TIP41*	*H2A*	*β-TUB*	*CDC2*
NormFinder	*DnaJ*	*α-TUB2*	*TIP41*	*GAPDH*	*H2A*	*α-TUB1*	*β-TUB*	*ACT*	*CDC2*
Delta CT	*ACT*	*α-TUB1*	*DnaJ*	*GAPDH*	*α-TUB2*	*TIP41*	*H2A*	*β-TUB*	*CDC2*
BestKeeper	*ACT*	*DnaJ*	*α-TUB1*	*α-TUB2*	*GAPDH*	*TIP41*	*H2A*	*β-TUB*	*CDC2*
Comprehensive	*ACT*	*DnaJ*	*α-TUB1*	*GAPDH*	*α-TUB2*	*TIP41*	*H2A*	*β-TUB*	*CDC2*
Ranking order under male flower bud (Better-Good-Average)									
geNorm	*ACT/DnaJ*		*α-TUB1*	*α-TUB2*	*GAPDH*	*TIP41*	*H2A*	*β-TUB*	*CDC2*
NormFinder	*GAPDH*	*α-TUB2*	*α-TUB1*	*TIP41*	*H2A*	*DnaJ*	*GAPDH*	*β-TUB*	*CDC2*
Delta CT	*ACT*	*DnaJ*	*α-TUB1*	*α-TUB2*	*GAPDH*	*TIP41*	*H2A*	*β-TUB*	*CDC2*
BestKeeper	*ACT*	*DnaJ*	*α-TUB1*	*α-TUB2*	*GAPDH*	*H2A*	*TIP41*	*β-TUB*	*CDC2*
Comprehensive	*ACT*	*DnaJ*	*α-TUB1*	*α-TUB2*	*GAPDH*	*TIP41*	*H2A*	*β-TUB*	*CDC2*
Ranking order under total samples (Better-Good-Average)									
geNorm	*DnaJ/TIP41*		*H2A*	*ACT*	*α-TUB1*	*α-TUB2*	*GAPDH*	*β-TUB*	*CDC2*
NormFinder	*ACT*	*DnaJ*	*TIP41*	*H2A*	*α-TUB1*	*β-TUB*	*α-TUB2*	*GAPDH*	*CDC2*
Delta CT	*ACT*	*DnaJ*	*TIP41*	*α-TUB2*	*H2A*	*α-TUB1*	*GAPDH*	*β-TUB*	*CDC2*
BestKeeper	*ACT*	*DnaJ*	*α-TUB1*	*GAPDH*	*α-TUB2*	*TIP41*	*H2A*	*β-TUB*	*CDC2*
Comprehensive	*ACT*	*DnaJ*	*TIP41*	*H2A*	*α-TUB1*	*α-TUB2*	*GAPDH*	*β-TUB*	*CDC2*

## Data Availability

Data is contained within the article or supplementary material.

## Supplementary Materials

The following supporting information can be downloaded at: https://www.mdpi.com/article/10.3390/plants11050647/s1, Figure S1: 1% agarose gel electrophoresis shows clear RNA bands, Figure S2: 1% agarose gel electrophoresis shows specific bands of PCR products, Figure S3: Melting curves of the nine reference genes showing single peaks, Figure S4: Amplification plots of the nine candidate reference genes.
