# Supplementary material for: A Selection of Reliable Reference Genes for Gene Expression Analysis in the Female and Male Flowers of Salix suchowensis
Source: Plants (Basel). 2022 Feb 27;11(5):647. doi: 10.3390/plants11050647 (PMC8912643; doi:10.3390/plants11050647)
Supplement: Supplementary file 1 [file plants-11-00647-s001.zip › plants-1573711-supplementary.pdf]

plants

MDPI

Supplementary Materials

Selection of Reliable Reference Genes for Gene Expression Analysis of Female and Male Flowers of *Salix suchowensis*

Fangwei Zhou, Yingnan Chen, Huaitong Wu * and Tongming Yin

Key Laboratory for Tree Breeding and Germplasm Improvement, Southern Modern Forestry Collaborative Innovation Center, College of Forestry, Nanjing Forestry University, Nanjing, 210037, China; 2540282810@qq.com (F.Z.), chenyingnan@njfu.edu.cn (Y.C.), tmyin@njfu.com.cn (T.Y.)
* Correspondence: wuhuaitong@163.com; Tel.: +86-150-5052-1502

A

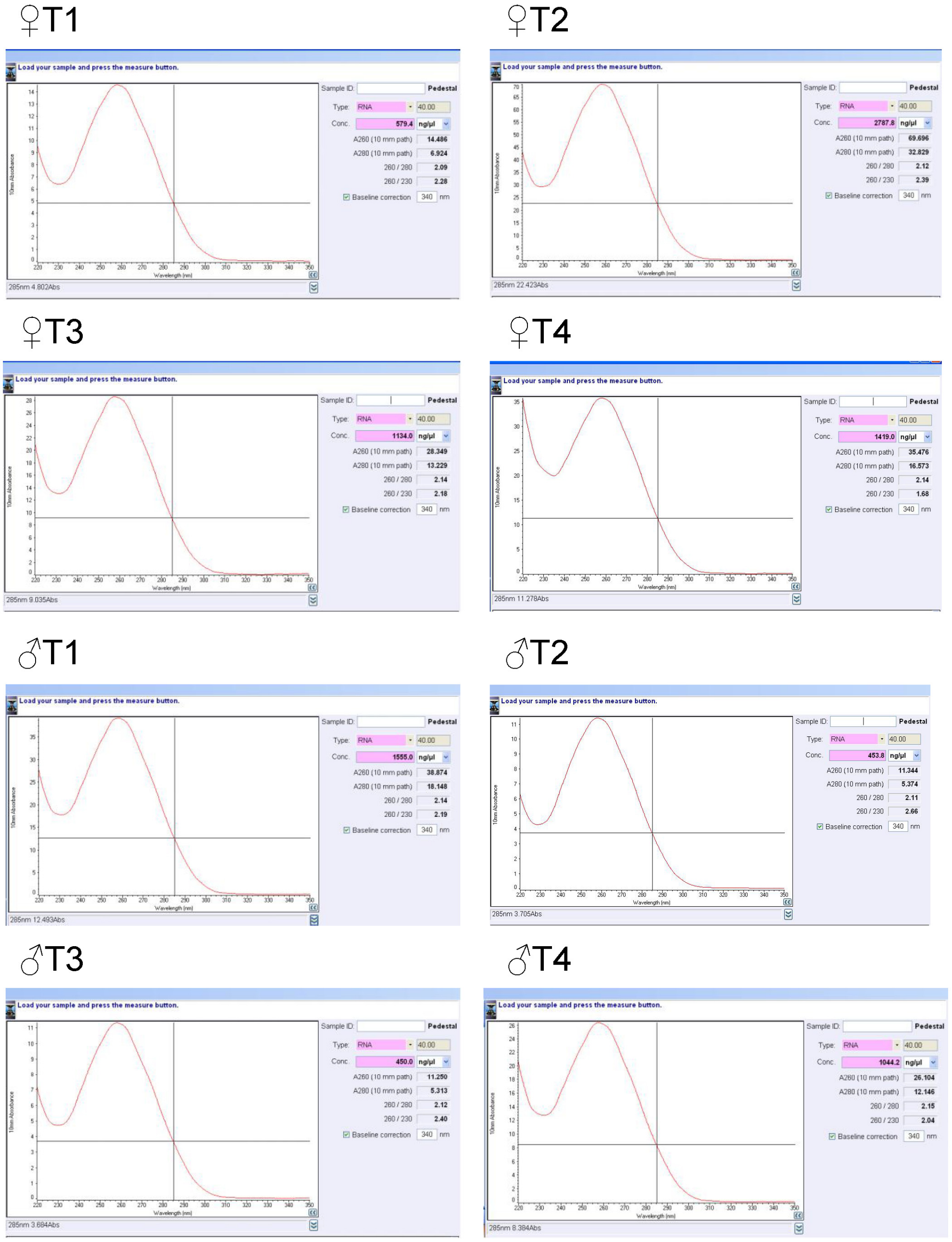

B

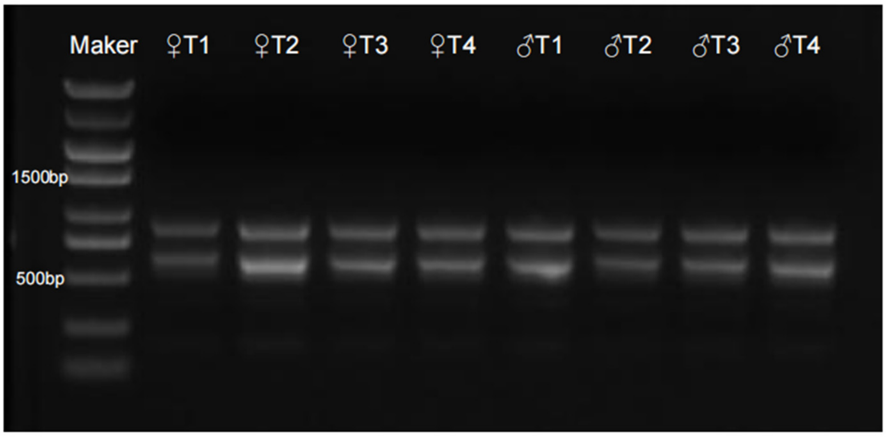

Figure S1. RNA concentration and quality assay. (A) NanoDrop™ One detects good RNA quality. (B) 1% agarose gel electrophoresis shows clear RNA bands.

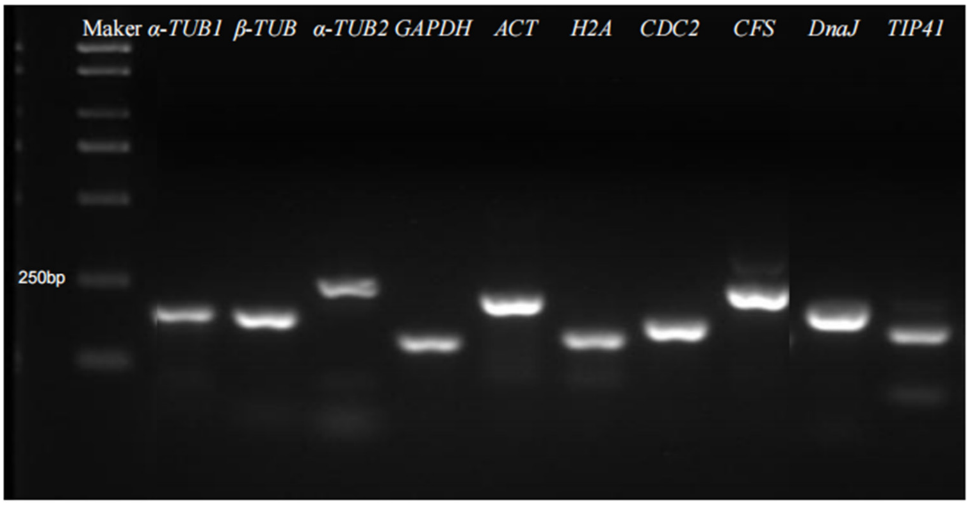

Figure S2. 1% agarose gel electrophoresis shows specific bands of PCR products.

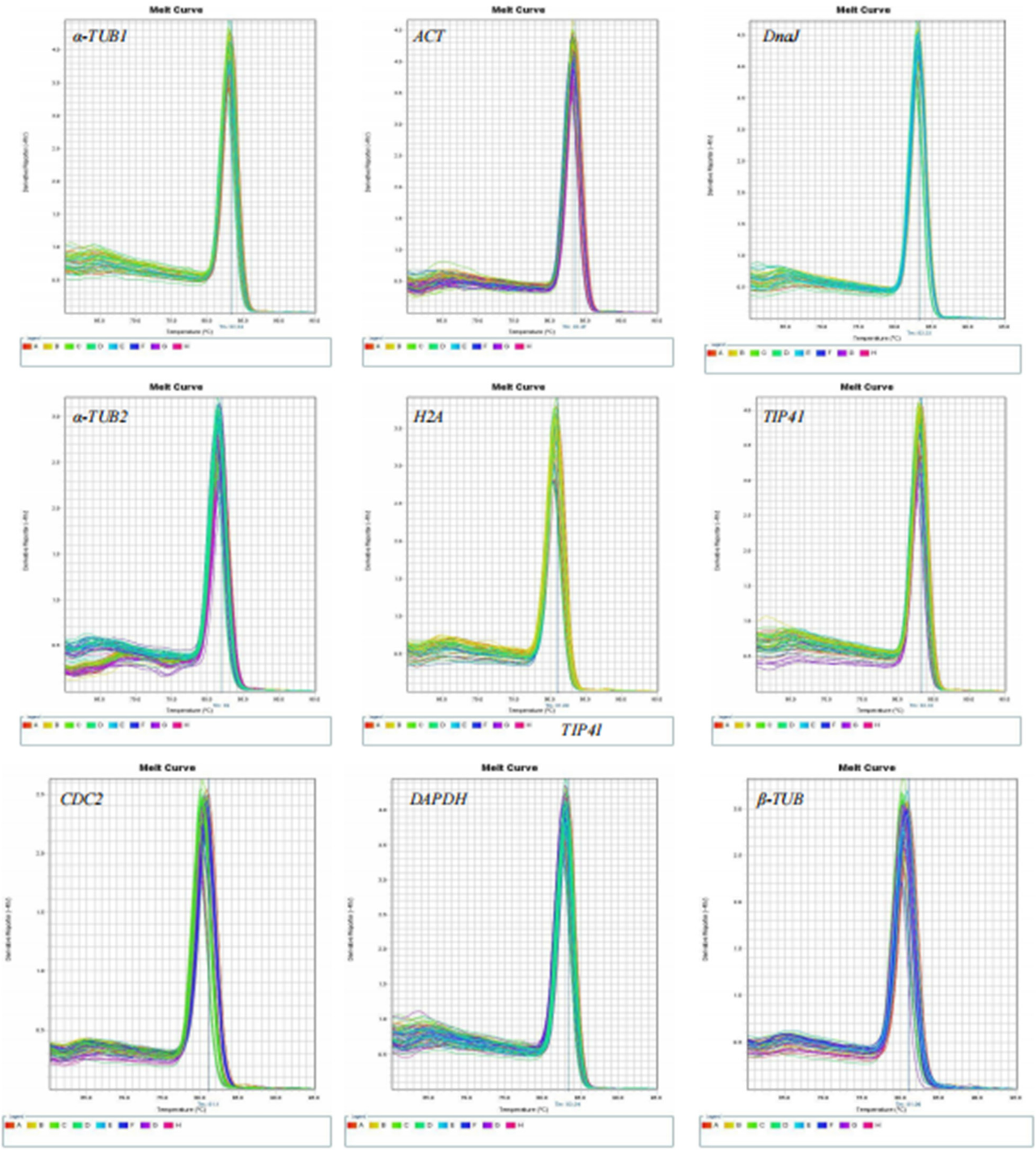

Figure S3. Melting curves of nine reference genes showing single peaks.

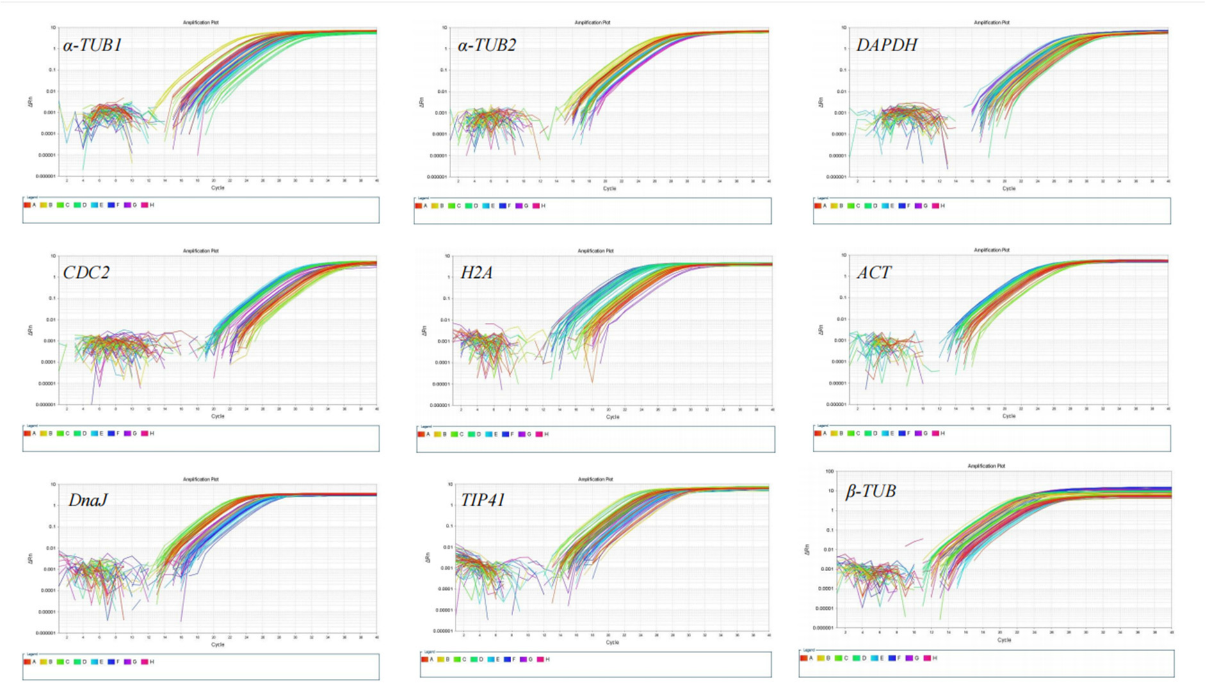

Figure S4. Amplification plots of the nine candidate reference genes.
